# Mesoporous Materials for Metal-Laden Wastewater Treatment

**DOI:** 10.3390/ma16175864

**Published:** 2023-08-27

**Authors:** Dmitrii Grozdov, Inga Zinicovscaia

**Affiliations:** 1 Department of Nuclear Physics, Joint Institute for Nuclear Research, Joliot-Curie Str., 6, 1419890 Dubna, Russia; dsgrozdov@rambler.ru; 2 Department of Nuclear Physics, Horia Hulubei National Institute for R&D in Physics and Nuclear Engineering, 30 Reactorului Str. MG-6, 077125 Magurele, Romania; 3 Institute of Chemistry, Moldova State University, 3, Academiei Str, MD-2028 Chisinau, Moldova

**Keywords:** mesoporous materials, adsorption, metals, wastewater, bioremediation, reusability

## Abstract

Rapid technological, industrial and agricultural development has resulted in the release of large volumes of pollutants, including metal ions, into the environment. Heavy metals have become of great concern due to their toxicity, persistence, and adverse effects caused to the environment and population. In this regard, municipal and industrial effluents should be thoroughly treated before being discharged into natural water or used for irrigation. The physical, chemical, and biological techniques applied for wastewater treatment adsorption have a special place in enabling effective pollutant removal. Currently, plenty of adsorbents of different origins are applied for the treatment of metal-containing aqueous solution and wastewater. The present review is focused on mesoporous materials. In particular, the recent achievements in mesoporous materials’ synthesis and application in wastewater treatment are discussed. The mechanisms of metal adsorption onto mesoporous materials are highlighted and examples of their multiple uses for metal removal are presented. The information contained in the review can be used by researchers and environmental engineers involved in the development of new adsorbents and the improvement of wastewater treatment technologies.

## 1. Introduction

Water contamination is one of the most significant issues the globe has faced in recent years. According to the United Nations, approximately 80% of all industrial and urban wastewater is discharged into the environment, particularly in developing countries [1], often without any treatment. Among the wide variety of pollutants entering the environment, heavy metals are one of the most dangerous due to their toxicity, persistence, and biomagnification [2]. Some elements, belonging to the heavy metals Co, Mo, Se, Cu, Cr, Fe, and Mn, perform important biochemical and physiological functions in living organisms, and their deficiency can result in numerous disorders or syndromes. At the same time, it is important to know that for elements such as, for example, Cr, Se, and Cu, there is a very narrow range of concentrations between beneficial and toxic effects [3]. 

The intensification of human activities and the active application of metals in agriculture, the plating and electroplating industry, the chemical industry, mechanical engineering, the textile industry, metal smelting, the petrochemical industry, etc. [2,3], has considerably increased the release of metals such as Al, Pb, Sb, Hg, As, and Cd, which have no biological functions and are toxic even at 1–100 µM levels, into natural water bodies [3,4]. Gastrointestinal and renal failure, neurological disorders, skin lesions, vascular damage, immune system problems, birth deformities, and cancer are a few outcomes brought on by the harmful effects of heavy metals. Metal ions also provoke the generation of reactive oxygen species and oxidative stress as well as enzyme inactivation [5,6,7,8]. 

Great efforts are required to reduce the negative impact of toxic elements on natural ecosystems. One of the important tools for addressing the problem is the careful treatment of wastewater before its discharge into the environment or further use. Currently, a wide variety of techniques, including biological treatment, coagulation, ultrafiltration, coagulation, flocculation, membrane separation procedures, chemical precipitation, ion exchange, enhanced oxidation, reverse osmosis, membrane filtration, ion exchange, electrochemical treatment, irradiation, extraction, and adsorption are applied to reduce concentrations of heavy metals in wastewater to maximum admissible levels [1,9,10]. However, the numerous drawbacks associated with these techniques, such as high cost, energy consumption, the frequent need for reagents, the unpredictable removal of heavy metal ions, and the production of hazardous sludge, make them unpopular [9,11]. For instance, precipitation produces a large amount of sludge, while membrane filtration, ion exchange, electro-deposition, and filtration are expensive techniques [12].

Adsorption has demonstrated great potential among the technologies available for metal removal due to its economic feasibility, ease of handling, the accessibility of sorbents, affordability, large surface area, high adsorption capacity, and environmental sustainability, as well as its potential selectivity for the target metal and low sludge generation. Heavy metal ions can be captured and removed from wastewater using commercial and natural adsorbents, which are often characterized by high removal capacity [13,14,15,16,17]. Nowadays, metal–organic frameworks [18,19], anionic clays [20,21], nanomaterials [22,23], activated carbon [24,25], hydroxyapatites [26,27], and natural minerals [28,29] are applied for wastewater treatment. It is worth mentioning that some drawbacks of these materials have already been identified, including their frequently low surface area and water stability as well as low adsorption capacity. Therefore, the design of new porous adsorbent materials with enhanced adsorption capabilities and great structural robustness is still necessary for the development of efficient remediation systems [4]. 

Functional mesoporous materials are now seen as intriguing compounds for a variety of applications, including drug delivery, biomedicine, catalysis, and the adsorption of pollutants of different origins. These materials are characterized by large pore volumes, exceptionally large specific surface area, homogeneous pore size distributions, and tunable pore sizes, which contribute to their technological advantages [30,31]. Additionally, adding appropriate functional groups to the surface can increase their affinity for the target metal ions [32].

The aim of the present review is to discuss the main researchers’ achievements in the synthesis and application of mesoporous materials for metal removal from aqueous solutions attained in the last several years. Furthermore, a discussion of the factors affecting the adsorbents’ removal capacity usually described in review papers [32,33], the techniques applied for mesoporous adsorbents’ production, and possible mechanisms of the adsorption are elucidated. Attention was also paid to the possibility of adsorbents’ multiple uses and the estimation of their cost.

## 2. Mesoporous Materials Synthesis and Characterization

The synthesis of mesoporous materials (pore diameters 2–50 nm) is a rapidly developing field of bioremediation studies. Today, mesoporous materials with different mesostructures and high surface areas can be obtained in different forms for a wide range of applications [34]. Recent techniques used to synthesize mesoporous materials include hydrothermal and microwave synthesis as well as sol–gel, phase conversion, and templating methods [35]. Hydrothermal methods have been extensively applied for the fabrication of mesoporous materials. These techniques allow the production of materials with high thermal and chemical stability, improved mesoscopic regularity, and extended pore sizes [36]. The sol–gel method, a chemical technique, is characterized by its simplicity and ability to produce materials with different morphologies [37,38]. The microwave-assisted synthesis method’s advantages include low energy consumption and the production of materials of uniform size [39]. Templating methods allow for precise control of the material’s particle morphology, pore diameter, and mesoporosity [40]. Regardless of their merits, most of the presented techniques are time-intensive, and some of them require the use of hazardous chemicals, including organic solvents, which can increase the price of the materials and have a negative impact on the environment [41,42,43].

The synthesis of new mesoporous materials requires their detailed characteristics in order to determine the particle size, pore morphology, surface area, and structure, and a detailed study of the surface of the produced material, including the specification of functional groups. A set of techniques, including X-ray diffraction (XRD), transmission and scanning electron microscopy (TEM and SEM), energy dispersive X-ray analysis (EDXA), Fourier transform infrared spectroscopy (FTIR), nitrogen adsorption–desorption, and thermal gravimetric analysis (TGA) can be applied to solve this task [44]. 

Among various mesoporous adsorbents available for the removal of heavy metals, silica-based materials are one of the most popular due to their large surface area and excellent chemical, thermal, and mechanical stabilities [17]. An introduction to the essential features of silicate mesoporous materials is provided in Alothman’s [45] review. The study includes an overview, a succinct historical introduction, a quick introduction to the science of surfactants, a thorough introduction to the science of sol–gels, a comprehensive review of MCM-41 modification techniques, and a synopsis of specific uses of these materials.

**Figure 1:**
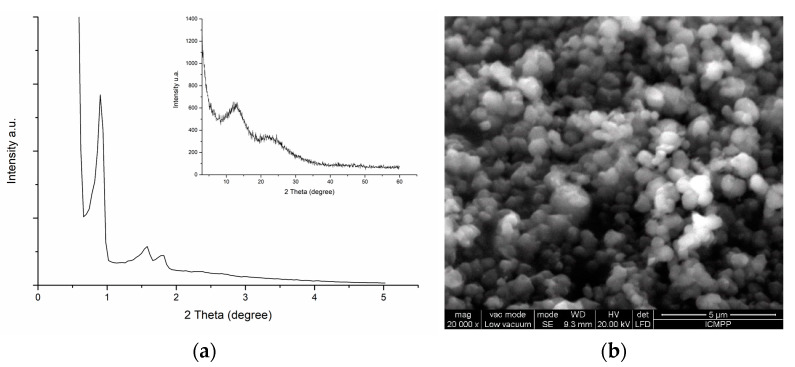
(**a**) XRD pattern and (**b**) SEM image of silica SBA-15 material [47].

The synthesis of SBA-15 consists of the dissolution of amphiphilic triblock copolymer in acids, followed by the addition of the silica source and further hydrothermal treatment [46]. The specified procedure was used in [47] to produce SBA with a mean pore diameter of 9 nm and a surface area of 802.493 m^2^/g. The XRD pattern confirmed the SBA-15 structure (Figure 1a) for synthesized material, and SEM images (Figure 1b) showed characteristic spherical particle morphologies of 400–500 nm in diameter. The FTIR analysis of the SBA-15 material revealed the stretching bonds of Si–O–Si, Si-OH, Si–O–Si and C=O groups [48]. 

Zhai [49] applied the hydrothermal method to synthesize SBA-15. TEM analysis showed clear and ordered pore stripes, indicating the mesoporous nature of the synthesized material. The pore size of the material amounted to 11.02 nm, and the specific surface area was 503 m^2^/g. The synthesis of SBA-15 material from the ash of a brickyard under strongly acidic conditions was reported by [50]. The surface area of the new mesoporous material was 700.1 m^2^/g, the pore volume was 0.813 cm^3^/g, and the pore size was 7.5 nm The typical XRD pattern for SBA-15 was obtained, while TEM showed the formation of parallel, uniform channels with uniform pore sizes. Kumar and co-authors [51] applied coal fly ash to produce porous materials, Al-MCM-41 and SBA-15, with hexagonal structures. The surfactant used in the synthesis of Al-MCM-41 was C_16_H_33_ (CH_3_)_3_NBr(CTAB), while SBA-15 was produced using (EO)_20_(PO)_70_(EO)_20_. The XRD pattern of MCM-41 showed the hexagonal structure of the material, while in the case of SBa-15, the excellent textural uniformity of the material was demonstrated. The surface area of Al-MCM-41 was 842 m^2^/g, the pore volume was 0.75 cm^3^/g, and the pore diameter was 3.7 nm. The surface area of SBA-15 was 483 m^2^/g, the pore volume was 0.53 cm^3^/g, and the pore diameter was 5.5 nm. The TEM images confirmed the pore system symmetries for Al-MCM-41 and showed the porous structure of SBA-15. 

Besides silica, mesoporous carbon-based and iron oxide-based materials can be applied for metal removal from wastewater. In the review by [33], the synthesis of different mesoporous carbon-based materials is presented in detail. The surface area of mesoporous iron oxide (MI) synthesized by applying a supramolecular templating method was 269 m^2^/g and a pore size of 6.9 nm. XRD measurements showed that the obtained MI was hematite (α-Fe_2_O_3_), and TEM images revealed that MI has an irregular shape with diameters in the 0.5–1 μm range [52]. A surfactant-templating sol–gel approach was used to prepare superparamagnetic microspheres with an Fe_3_O_4_@SiO_2_ core and a perpendicularly aligned mesoporous SiO_2_ shell [53]. The XRD pattern revealed hexagonal mesopore symmetry.

Metal–organic frameworks (MOFs), which are becoming an alternative to traditional inorganic porous materials, are limited in their application in metal removal by the weak metallic cation interactions due to the low metal ion constants. However, the functionalization of MOFs enables them to interact strongly with metal ions [54]. MOF-5 adsorbent produced by a solvothermal method had a specific surface area of 500.8 m^2^/g and a total pore volume of 0.19 cm^3^/g [55]. The XRD pattern showed the high crystallinity of the synthesized compound, while SEM images showed a wide number of spherical particles arranged in irregular assemblies. The FTIR analysis demonstrated the presence of hydroxyl, amine, carboxyl, and C-H groups. Nqombolo and co-authors [56] reported the synthesis of a cobalt/zinc-based MOF (ZIF-67/ZIF-8) through a facile method. The obtained adsorbent had a surface area of 950 m^2^/g and an average pore size of 2.88 nm. A Zn-based metal–organic framework, Zn_2_(DPTTZ) (OBA)_2_ (IUST-2), was synthesized through a solvothermal method. The specific surface area of the IUST-2 was 105.636 m^2^/g and the pore size was 3.0 nm. According to XRD analysis, the produced adsorbent is an orthorhombic crystal system [57].

Often, the adsorption capacity of the neat mesoporous materials toward metal ions is low. The functionalization of the surface of mesoporous materials with organic or inorganic functional groups leads to new physical and chemical properties [45] and can enhance the adsorption capacity toward heavy metals [58]. Thus, [59] showed that among a series of adsorbents of SBA-15-type with different ligands, neat SBA-15 showed the lowest adsorption capacity toward rare earth elements and heavy metals. 

A facile synthesis of functionalized mesoporous silica nanoparticles (MSN) including the polymerization of N-isopropylacrylamide (NIPAM) monomer with vinyl functional mesoporous silica nanoparticles (VMSN) was proposed by [30]. The surface area of the synthesized mesoporous material was 1085 m^2^/g and the pore diameter was 2.5 nm. The XRD pattern and SEM indicated the formation of highly ordered 2D hexagonal mesostructures with particle sizes in the range of 80 nm and 350 nm. The Si–O–Si, Si–OH, C–H and Si–C peaks were seen on the material FTIR spectrum. Mesoporous silica material, MCM-41, was prepared by applying a microwave heating technique and using silica fume as a silica source. Microwave heating allowed a significant reduction in the time required for MCM-41 synthesis. The surface area of the obtained adsorbents ranged from 1253 to 1633 m^2^/g, the pore volume ranged from 0.16 to 0.65 cm^3^/g, and the pore diameter was 3.6 nm for all sorbents, except h-MCM-41(40), for which it was 3.3 nm. Compared to samples made with hydrochloric acid, samples produced with citric acid showed a greater mesoporous structure [31]. A composite of MCM-41 silica with rice husk was produced using a hydrothermal method. SEM images showed the deposition of the silica material on the rice husk surface, while TEM proved the hexagonal mesostructure of the material [60]. The synthesis of 2-acetylthiophene-modified SBA-15 mesoporous material (A-SBA-15) was performed in three steps. The first two steps included the synthesis of the SBA-15 and its functionalization with –NH_2_ groups, while in the third step, the obtained material was modified with 2-acetylthiophene. As a result, a material with a uniform pore size of 8.1 nm (TEM results) and a surface area of 335.6 m^2^/g was obtained [61].

The preparation of conjugate material by direct immobilization of functional organic ligand 6-((2-(2-hydroxy-1-naphthoyl)hydrazono) methyl)benzoic acid onto porous silica was described by [62]. The TEM micrographs showed that the prepared material exhibits a typical hexagonal structure with ordered mesoporous frameworks. Magnetic mesoporous silica (NZVI-SH-HMS) immobilized with thiol and nanometre zero-valent iron was produced by combining gel–sol and wet impregnation techniques. The material possessed good porous properties with a specific surface area of 312.84 m^2^/g and a proper pore size of 2.56 nm. XRD showed that the material was superparamagnetic, while, using SEM, the regular spherical structure with mesoporous channels was seen [63]. Mesoporous silica Cl-SBA-15 or Cl-MCM-41 was obtained by the immobilization of the -chloropropyltriethoxysilane on the mesoporous silica surface. Next, 2-mercaptothiazoline, MTZ was introduced to obtain the functionalized silicas defined as MTZ-SBA-15-Het or MTZ-MCM-41-Het. The surface areas of the obtained materials were 740 m^2^/g and 1172 m^2^/g, and the average pore diameters were 7.2 and 3.4 nm, respectively [64]. A mesoporous organosilica adsorbent DAPy@MSA NPs synthesized using sol–gel co-condensation, as reported by [65], had a surface area of 465 m^2^/g and a pore size of 4.4 nm. The synthesis of Fe_3_O_4_@SiO_2_@m-SiO_2_–NH_2_ by coating the as-synthesized magnetic cores with a silica shell in the first stage and the application of a cationic surfactant as a structure-directing agent in the second stage is described in [66]. The obtained sorbent was characterized by strong magnetization, a surface area of 637.38 m^2^/g, and a pore size of 2.85 nm. The XRD analysis indicated the face-centred cubic configuration of the magnetic adsorbent. The functionalization of Fe_3_O_4_@nSiO_2_@mSiO_2_ with EDTA resulted in an obtained material with a pore diameter of 2.1 nm and a surface area of 337.02 m^2^/g [67]. The hexagonal mesoporous structure of the material was proven by XRD analysis. The FTIR spectra showed the typical vibrations of carboxylic and C–N groups. Peng and co-authors [68] applied the co-precipitation technique for the synthesis of nano-Fe_3_O_4_-modified high-iron red mud (HRM@nFe_3_O_4_). The specific surface area of the produced material was 171.63 m^2^/g, with an average pore size of 7.62 nm and a pore volume of 31 cm^3^/g. 

Thus, by applying different approaches, a wide variety of materials with adsorption properties can be produced. Table 1 contains additional examples of mesoporous material preparation.

## 3. Application of Mesoporous Materials for Metal Removal from Wastewater

Da’na’s work [32] summarized the main researchers’ contributions to the adsorption of heavy metals on functionalized mesoporous silica carried out before 2017. Thus, the present review is mainly focused on the research performed in the period 2018–2023. Metal adsorption is a process dependent on many factors, including experimental conditions such as the acidity of the solution, contact time, temperature, and adsorbate concentration, as well as the adsorbent’s affinity to adsorbate [89]. The description of the kinetic and equilibrium models applied to explain the adsorption process is presented in many review papers [90,91,92,93].

The ability of mesoporous bifunctional magnetic NZVI-SH-HMS to remove Cd and Pb from solutions was investigated. Low adsorption at pH 3.0 was replaced by a drastic increase with the increase in pH from 3.0 to 4.0, indicating that the removal process was pH-dependent. For both elements, adsorption equilibrium was reached in a short time: 30 min for Pb and 50 min for Cd, and the kinetics of the process were presented by a pseudo-second-order model. Adsorption was better described by the Langmuir model with a maximum adsorption capacity of NZVI-SH-HMS 487.8 mg/g for Pb and 330.0 mg/g for Cd. Additionally, NZVI-SH-HMS demonstrated good adsorption capacity and sorption recyclability in the case of real wastewater of different origins [63]. Zhai [49] studied the effect of experimental conditions on Cu removal by the SBA-15 molecular sieve. The maximum adsorption capacity of 11.39 mg/g was achieved under optimized adsorption conditions (pH 3.5, adsorbent dosage 0.0050 g, time 40 min). It was shown that the kinetics of the process were described using the pseudo-second-order kinetic model, while equilibrium applied the Freundlich model. From a thermodynamic point of view, the process was spontaneous and exothermic. At the same time, Knight et al. [94], studying the nano-scale confinement effects on the adsorption of Cu on mesoporous silica with pore sizes of 8, 6, and 4 nm showed low metal adsorption onto the surface of SBA-15, with the maximum measured surface loading of 0.020 ± 0.001, 0.019 ± 0.002, and 0.039 ± 0.002 μmol/m^2^ for SBA-15-8, SBA-15-6, and SBA-15-4, respectively. The effect of contact time and pH on Zn adsorption onto SBA-16 and SBA-15 modified with APTES (3-aminopropyltriethoxy-silane) and then with EDTA was investigated by [95]. For both adsorbents, equilibrium was reached within the first 30 min and maximum removal was obtained at pH 6.0. Equilibrium data were better described by the Freundlich isotherm model and the maximum adsorption capacity amounted to 184.1 mg/g for SBA-16 and 108 mg/g for SBA-15.

The removal of As(III) and As(V) using amidoxime resin embedded into mesoporous silica was highly affected by the solution pH. The maximum removal of As(V) was attained at pH 3.0 and of As(III) at pH 8.0. The time required for equilibrium attainment was 3–4 h. The maximum adsorption calculated from Langmuir models constituted 3.8 mmol/g for As(III) and 3.1 mmol/g for As(V) [96]. EDTA-modified magnetic mesoporous microspheres (Fe_3_O_4_@nSiO_2_@mSiO_2_/EDTA) showed high adsorption capacity for Cr(III), and the maximum adsorption of 30.59 mg/g was obtained at pH 3.0 and 25 °C. The equilibrium data fitted well to the Freundlich isotherm model. Studying the effect of cations (Na^+^, K^+^ and Ca^2+^) and complex agents (EDTA, citric acid and formic acid), the authors showed that the adsorption capacity of Fe_3_O_4_@nSiO_2_@mSiO_2_/EDTA for Cr(III) did not change significantly [67]. The Fe_3_O_4_@SiO_2_@m-SiO_2_ microspheres showed a high sorption capacity of 834.18 mg/g for Cd at pH 6.0 after 20 min of contact. The adsorbent maintained high adsorption capacity during six sorption–desorption cycles [66]. Antimony adsorption on nano-Fe_3_O_4_-modified high-iron red mud (HRM@nFe_3_O_4_) was not dependent on the pH of the solution. The maximum adsorption capacity of Sb(III) on HRM@nFe_3_O_4_ computed from the Langmuir model was 98.03 mg/g, while both the pseudo-first-order and the pseudo-second-order models were suitable for the explanation of the experimental data. Studying the effect of co-existing ions, it was shown that Na^+^, NH_4_^+^, K^+^, Ca^2+^, Mg^2+^, SO_4_^2−^, and Cl^−^ did not affect Sb(III) adsorption; however, it was inhibited by the presence of SiO_3_^2−^ and PO_4_^3−^ [68]. A set of SBA-15 adsorbents functionalized with ethylenediaminetriacetic acid, primary amine, and quaternary ammonium were applied for Cr(III, VI), Mn(II, VII), Pb, Cd, and Cu adsorption. The adsorbent functionalized by ethylenediaminetriacetic acid showed the highest adsorption capacity for the studied metal ions, which amounted to 195.6 mg/g for Pb, 111.2 mg/g for Cd, 57.7 mg/g for Cr(III), 58.7 mg/g for Cu, and 49.4 mg/g for Mn(II). The presence of organic matter and major water cations did not affect the efficiency of the studied metal ions’ removal. The maximum adsorption for all studied cations was attained at pH 4.0–6.0, while elements in anionic form were more efficiently removed in the pH range from 5.0 to 8.0. The efficiency of metals’ removal from artesian, urban river, and lake water was at the level of 96% for all metal ions [97].

A new Zn-based MOF (IUST-2), showed maximum Pb and Hg adsorption at pH values of 5.0 and 4.0, respectively. The theoretical maximum adsorption of Pb and Hg ions was computed to be 1430 and 900 mg/g, respectively [57]. The maximum adsorption capacity of the Cu–MOF toward Cd at 219.05 mg/g was attained at pH 4.0, a contact time of 60 min, and an adsorbent dosage of 0.5 g [82]. Nabipour et al. [98] discussed in detail the use of MOFs for Cd removal from wastewater, while Shellaiah and Sun [99] described their application for mercury removal. The guidance for the synthesis of novel MOF adsorbents and their application for U adsorption was provided by [100].

More examples of mesoporous materials’ applications for metal removal are presented in Table 2.

## 4. Mechanisms of Metal Ions’ Removal by Mesoporous Materials

**Figure 2:**
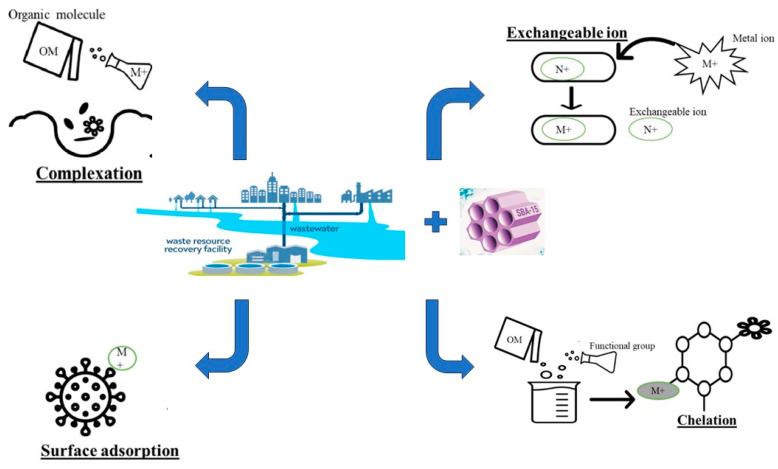
The scheme of possible mechanisms involved in metal ions removal by mesoporous adsorbents.

The adsorption of heavy metals can proceed in several ways, and the most important mechanisms could be the van der Waals interaction, ion exchange, hydrogen bonding, complexation, and precipitation [89]. The scheme of the possible interactions of metal ions with mesoporous materials is presented in Figure 2.

Zhu et al. [31] suggested that Cu, Pb, and Cd adsorption onto c-MCM-41(40) occurred in three steps: (i) external surface adsorption; (ii) gradual adsorption, when intraparticle diffusion into the mesopores plays the dominant role; and (iii) the final stage, characterized by the reduction of the intraparticle due to the low metal ions’ concentration in the solution. Studying Pb and Cd removal by NZVI-SH-HMS, the authors showed the involvement of –OH and –SH groups in Pb removal as well as the formation of Pb_3_(CO_3_)_2_(OH)_2_ and PbS. In the case of Cd removal, it mainly took place due to its precipitation in the form of hydroxide or sulphide and co-precipitation with Fe^2+^ in the material and free hydroxyl in solutions [63]. The adsorption of Hg and Pb onto IUST-2 took place mainly due to metal ions’ interaction with S and N atoms of the thiazole ring [57].

Copper ions’ adsorption onto DAPy@MSA NPs occurred mainly due to the coordination of Cu ions with the pyridyl and hydroxyl groups [65]. Da’na and Sayari [109] presented the mechanism of Cu adsorption onto functionalized SBA-15. The authors suggested that, firstly, Cu ions interacted with amine groups, and the [Cu(RNH_2_)_2_]^2+^ complex was formed. As the Cu concentration in solution increases, the [Cu(RNH_2_)_2_]^2+^ turns into [Cu(RNH_2_)]^2+^, enhancing adsorption. The reaction of Cu with hydroxyl groups via Si–O–Cu–O–Si bridging species is considered another possible mechanism of metal ion removal. The adsorption of Cu onto SBA-1 occurred due to the formation of a covalent bond between the Cu atom and oxygen in the adsorbent medium, Si–O–Cu [103]. The OH and –NH_2_ groups as well as p-delocalized electrons of the triazine ring (C_3_N_3_) participated in Cu adsorption onto Zr–G–C3N4 sorbent [110]. At the same time, studying Cu adsorption on several modifications of SBA-15, the authors observed a lack of interaction between Cu ions and OH groups. The adsorption process occurred mainly due to electrostatic interactions and the formation of dimeric species adsorbed onto the amorphous silica surface was noticed [94]. Ryu et al. [58] showed that Cu adsorption on SBA-15 is possible due to amine groups’ complexation with Cu ions.

The mechanism responsible for Hg adsorption onto ethylenediamine functionalized KIT–6 (DA-KIT–6) silica materials was physical adsorption, which consisted of the formation of a physical bond between the Hg ions and basic nitrogen atoms on the adsorbent surface [108]. It was proposed that As(V) adsorption onto mesoporous iron oxide is due to electrostatic forces between As(V) and the adsorbent, while that of As(III) is due to van der Waals attraction [52]. The adsorption of Cr(VI) onto α-FeOOH consisted of both chemical and physical adsorption involving electrostatic attractions. It should be mentioned that more than 61.7% of Cr was adsorbed in the form of Cr(III) and the rest in the form of Cr(VI). This can be explained by the presence of hydroxyl and methyl groups on the adsorbent surface [87]. The removal of Cr(III) by EDTA-modified magnetic mesoporous microspheres (Fe_3_O_4_@n-SiO_2_@mSiO_2_/EDTA) proceeded by ion exchange and surface complexation mechanisms [67]. Electrostatic interaction between anionic Cr(VI) and NH_3_^+^ groups is a possible mechanism for Cr(VI) sorption by amino-functionalized mesoporous silica [88]. Electrostatic interactions and the p-anionic interaction that exists in the 2-imidazole ligand contributed to the adsorption of Cr(VI) and As(V) onto cobalt/zinc-based metal–organic frameworks [56]. The ion exchanges, electrostatic interaction, and physical sorption, including pore filling, participated in Cu, Ni, Cd, and Pb adsorption onto mesoporous HNT-BC@Alg adsorbent [110]. The Zn adsorption onto EDTA-modified SBA-16 and SBA-15 occurred mainly due to Zn ions binding to the carboxylate groups of EDTA [95]. The adsorption of Sb(III) onto HRM@nFe_3_O_4_ occurred mainly due to surface complexation between Sb(III) and amorphous ferric oxide. The presence of Fe^3+^ can contribute to additional Sb(III) removal by co-precipitation [68]. According to FTIR analysis, In adsorption onto SBA-15 occurred mainly due to its interaction with carboxylic and hydroxyl groups. Theoretical calculations performed by the authors showed that three covalent bonds can be formed between In atoms and the oxygen atoms of silanol groups on the surface of the adsorbent [48].

The mechanisms of metal ions’ removal by mesoporous materials are strongly dependent on the type of the applied adsorbent as well as experimental conditions. At the same time, metal ions’ interaction with functional groups can be specified as the most frequently reported mechanism of adsorption onto mesoporous materials.

## 5. Mesoporous Adsorbents Reusability and Cost

To justify the economic and environmental value of the adsorbent, it is important to demonstrate not only its high adsorption capacity but also the possibility of the adsorbent’s multiple uses. The repeated use of adsorbents allows for a reduction in the cost of the wastewater treatment process. In desorption experiments, an essential component is the eluent used for metal recovery, which should effectively elute the adsorbed heavy metals without altering the adsorption capacity of the adsorbent [97]. The attempts to elute metal ions were made using HCl, HNO_3_, EDTA, NaOH, etc. In some cases, depending on the type of adsorbent, specific eluents can be applied. For example, in the case of CeO_2_-based mesoporous adsorbents, the ion exchange mechanism with peroxide treatment can be applied for sorbent regeneration due to the reaction of CeO_2_ with H_2_O_2_, resulting in the formation of stable cerium-oxo and peroxide bonds on the CeO_2_ surface. This technology allowed for the recycling of the adsorbent for six consecutive adsorption–desorption cycles in the Othman et al. [111] study.

The adsorption capacity of amine-functionalized SBA-15 used for Cu adsorption after ten cycles was 90% comparable to the raw material when EDTA was used for regeneration [109]. Mesoporous iron oxide applied for As adsorption was efficiently regenerated under basic conditions and maintained high adsorption capacity after five sorption-desorption cycles [52]. NaOH applied as a desorbing agent easily regenerated the Zr–G–C3N4 nanocomposite applied for Cu adsorption during four adsorption–desorption cycles [110]. The uptake capacity of the functionalized SBA-15 adsorbents did not change in five to six regeneration cycles when using chelating agent treatment [97]. The mesoporous-activated carbon showed no significant loss of adsorption capacity after three cycles of reuse when it was used for Cu and Pb adsorption [85]. Mesoporous adsorbents, SBA-15-EDTA, and SBA-16-EDTA were regenerated using HCl solution for five cycles, maintaining their removal capacity on the level of 85% [95]. Mesoporous carbon applied for cobalt removal was efficiently regenerated using H_2_SO_4_, losing only 5% of its adsorption capacity [83]. Thus, mesoporous material can be reused for metal ion adsorption as long as its adsorption and desorption capacities differ only slightly from the first adsorption–desorption cycle.

The cost of adsorbent synthesis and application is an important factor that determines material suitability for wastewater treatment in light of existing competing technologies [112]. Kobylinska et al. [97] estimated that the price of the functionalized SBA-15 adsorbent synthesis will vary from 2.3 to 2.49 EUR per 1 g of adsorbent. The cost of 1 g of nanozeolite produced by Pham et al. [113] was 0.12 USD, however, the cost of the adsorbent required for the treatment of 1000 kg of wastewater was 264.6 USD. The overall cost of the adsorption unit, in which nanoscale zero-valent iron was used as an adsorbent, was 3.15 USD/m^−3^ at optimal experimental conditions [114].

For comparison, the price of one ton of mineral–organic hybrid adsorbent, consisting of bacteria *Shewanella xiamenensis* biofilm and zeolite was calculated to be 530 USD [115]. The authors estimated that the cost of one ton of single-walled carbon nanotubes, multiwalled carbon nanotubes, and granular activated carbon was 90,000, 12,000, and 1000 USD, respectively [116]. Mukherjee and co-authors [117] showed that the price of the activated carbon produced from spent coffee grounds is in the range of 150–280 USD per ton, depending on the production route. Thus, the price of the mesoporous materials was comparable to or lower than the price of other adsorbents applied for metal removal from wastewater.

## 6. Conclusions

Mesoporous materials are excellent candidates for the removal of heavy metals from wastewater. Their application in remediation processes is determined by the high variety of materials; the possibility of adsorbent modification, which allows for increasing their adsorption capacity; high removal efficiency; and resistance to aggressive media. Moreover, the mesoporous materials can be easily regenerated, and their adsorption capacity is maintained at a high level even after more than five cycles of adsorption–desorption. Several mechanisms, including physical adsorption, complexation, coordination, ion exchange, and precipitation participate in metal adsorption on the mesoporous materials. However, the main role belongs to metal ions’ interaction with functional groups. Consequently, further efforts of scientists and engineers should be directed toward the development of low-cost mesoporous materials for large-scale applications.

## Figures and Tables

**Table 1:** Synthesis of mesoporous materials.

Material	Technique Applied for Synthesis	Surface Area, m^2^/g	Pore Size, nm	Reference
Mesostructured zeolitic materials (MZMs)	Conventional hydrothermal treatment	210–814	2.2–10	[69]
Micro/mesoporous ZSM-5 zeolite	Hydrothermal crystallization route, using starch mesotemplate	-	5–15	[70]
Mesoporous ZSM-5 zeolite	Facile grinding synthesis method	77	3–25	[71]
Mesoporous TS-1 zeolite (MTS-1)	Hydrothermal method using polydiallyldimethylammonium chloride (PDDA) as mesopore template	61.79	5–20	[72]
SBA-15 mesoporous silica with incorporated titanium	One-pot hydrothermal crystallization method	472	6.2	[73]
Titanium dioxide-functionalized dendritic mesoporous silica	Post-grafting method	666.66.	22.2	[74]
Mesoporous cerium oxide	Calcination of basic cerous carbonate (as a precursor) obtained by precipitation from an aqueous solution	6–102	3–4	[75]
Mesoporous cerium oxide	Thermal hydrolysis method	190–205	27	[76]
Mesoporous titanium dioxide	Soft-template method with titanium isopropoxide as a titanium source	90	19.2	[77]
Bridged silsesquioxanes	Sol–gel condensation of bis [3-(trimethoxysilyl)propyl]amine and N- methyl-3,30-bis(trimethoxysilyl)dipropylamine in acidic media in the presence of surfactants.	18.7–189.4	10.2	[78]
Mesoporous aluminosilicate/zeolite composite	Template co-precipitation method	320	7	[79]
PABA-MCM-41 mesoporous material	Hydrothermal/co-condensation method	556	4.2	[80]
Ion-imprinted mesoporous silica	Co-condensation method with uranyl as the template ion and diethylphosphatoethyltriethoxysilane as the functional ligands	224–681	1.06–9.55	[81]
Cu-MOF	Solvothermal method	1057	20	[82]
Mesoporous carbon	Replica method	439–924	9–13	[83]
Mesoporous carbon microspheres	Spray-drying method	1061	9.5	[84]
Mesoporous activated carbon	Self-activation method	843.3	3.55	[85]
Mesoporous activated carbon	Chemical activation	688.2	3.2	[86]
Mesoporous α-FeOOH nanoparticles	Freeze-drying technique	46	11	[87]
(3-aminopropyl)trimethoxysilane functionalized mesoporous silica	Post-synthesis grafting method	857.88	2.7	[88]
Amino-functionalized mesoporous silica nanoparticles	Base-catalysed hydrolysis and condensation	517.4	8.94	[17]
NZVI-SH-HMS	Gel–sol and wet impregnation methods	312.84	2.56	[63]
HRM@nFe_3_O_4_	Co-precipitation method	171.63	22.76	[68]

**Table 2:** Metal adsorption on mesoporous adsorbents.

Sorbent	Metal	pH	q, mg/g	Isotherm Model	Surface Area, m^2^/g	Reference
Mesoporous iron oxide	As(III)	5–9	136.89	Freundlich	269	[52]
	As(V)	5–9	31.82	Langmuir		
Iron oxide nanoparticles immobilized on cellulose nanofibril aerogels	As(III)	7	48	Langmuir	165	[101]
	As(V)	7	91			
ZIF-67/ZIF-8	As(V)	6.5	71.4	Langmuir	950	[56]
	Cr(VI)	6.5	69.4			
MOF-5	Cr(VI)	2.0	78.12	Langmuir	500.8	[55]
Mesoporous α-FeOOH nanoparticles	Cr(VI)	3	16.58	Langmuir	46	[87]
(3-aminopropyl)trimethoxysilane functionalized mesoporous silica	Cr(VI)	3	89.4	Temkin	857.88	[88]
Amino-functionalized mesoporous silica nanoparticles	Cr(VI)	2.0	42.2	Langmuir	517.4	[17]
Mesoporous carbon microspheres	Cr(VI)	3.0	156.3	Langmuir	1061	[84]
Polypyrrole/hollow mesoporous silica particle	Cr(VI)		322	Langmuir	325	[102]
Multi-modified SBA-15 (Mn-SBA-15-NH2)	Cu	5.0	2.01 mmol/g	Langmuir	310	[58]
SBA-15 Silica	Cu	5.0	52.71	Langmuir	802.493	[103]
ETS-10 titanosilicate	Cu	6.0	172.53	Langmuir	31.473	[103]
Mesoporous aluminosilicates	Cu	4.0	16	Langmuir	243	[104]
Mesoporous activated carbon	Cu	6.0	12	Langmuir	843.3	[85]
Mesoporous silica nanoparticles modified by dibenzoylmethane	Cu	6	31.76	Langmuir	-	[105]
Mesoporous carbon	Co	4.0–6.0	5.85	Langmuir	439–924	[83]
NZVI-SH-HMS	Cd	4.5	330.0	Langmuir	312.84	[63]
Mesoporous material (DMOS)	Cd	6.0	107	Langmuir	431	[106]
Mesoporous silica nanoparticles modified by dibenzoylmethane	Cd	6.0	35.37	Langmuir	-	[105]
PEI/MCM-41 *	Cd	6.0	156.0	Langmuir/Freundlich	440	[107]
Mesoporous silica nanoparticles modified by dibenzoylmethane	Hg	6.0	25.17	Langmuir	-	[105]
DA-KIT–6	Hg	10	50	Langmuir	185	[108]
SBA-15 Silica	In	6.0	2036	Langmuir	802.493	[48]
Mesoporous activated carbon	Pb	6.0	12.7	Langmuir	843.3	[85]
Mesoporous composite material	Pb	6.0	196.35	Langmuir	527	[62]
NZVI-SH-HMS	Pb	5.5	487.8	Langmuir	312.84	[63]
Mesoporous activated carbon	Zn	5.2	100.76	Langmuir	688.2	[86]
PEI/MCM-41 *	Ni	6.0	139.7	Langmuir/Freundlich	440	[107]

* Nano-spherical amine-rich polyethylenimine (PEI) grafted on mesoporous silica (MCM-41).

## Data Availability

Not applicable.
